# An Eye Tracking Study on the Perception and Comprehension of Unimodal and Bimodal Linguistic Inputs by Deaf Adolescents

**DOI:** 10.3389/fpsyg.2017.01044

**Published:** 2017-06-21

**Authors:** Eliana Mastrantuono, David Saldaña, Isabel R. Rodríguez-Ortiz

**Affiliations:** Departamento de Psicología Evolutiva y de la Educación, Universidad de SevillaSeville, Spain

**Keywords:** eye-tracking, deaf students, cochlear implants, native signers, discourse-level comprehension, sign-supported speech, peripheral vision

## Abstract

An eye tracking experiment explored the gaze behavior of deaf individuals when perceiving language in spoken and sign language only, and in sign-supported speech (SSS). Participants were deaf (*n* = 25) and hearing (*n* = 25) Spanish adolescents. Deaf students were prelingually profoundly deaf individuals with cochlear implants (CIs) used by age 5 or earlier, or prelingually profoundly deaf native signers with deaf parents. The effectiveness of SSS has rarely been tested within the same group of children for discourse-level comprehension. Here, video-recorded texts, including spatial descriptions, were alternately transmitted in spoken language, sign language and SSS. The capacity of these communicative systems to equalize comprehension in deaf participants with that of spoken language in hearing participants was tested. Within-group analyses of deaf participants tested if the bimodal linguistic input of SSS favored discourse comprehension compared to unimodal languages. Deaf participants with CIs achieved equal comprehension to hearing controls in all communicative systems while deaf native signers with no CIs achieved equal comprehension to hearing participants if tested in their native sign language. Comprehension of SSS was not increased compared to spoken language, even when spatial information was communicated. Eye movements of deaf and hearing participants were tracked and data of dwell times spent looking at the face or body area of the sign model were analyzed. Within-group analyses focused on differences between native and non-native signers. Dwell times of hearing participants were equally distributed across upper and lower areas of the face while deaf participants mainly looked at the mouth area; this could enable information to be obtained from mouthings in sign language and from lip-reading in SSS and spoken language. Few fixations were directed toward the signs, although these were more frequent when spatial language was transmitted. Both native and non-native signers looked mainly at the face when perceiving sign language, although non-native signers looked significantly more at the body than native signers. This distribution of gaze fixations suggested that deaf individuals – particularly native signers – mainly perceived signs through peripheral vision.

## Introduction

The current generation of deaf adolescents has benefitted from technological advances in hearing aids and cochlear implants (CIs), and from increasingly early diagnosis of hearing loss and intervention. Better access to auditory input and to the development of spoken language has raised doubts about the usefulness of combining linguistic input from signs with spoken language for implanted children (Knoors and Marschark, 2012; Spencer, 2016). A number of studies (McDonald Connor et al., 2000; Geers and Sedey, 2002; Geers et al., 2003; Spencer et al., 2003; Geers, 2006; Spencer and Tomblin, 2006; Miyamoto et al., 2009) have investigated the role of signs in enhancing spoken language development by comparing language skills of children with CIs exposed to spoken language-only in Oral Communication (OC) settings and children enrolled in Total Communication (TC) settings. TC includes all communicative systems where spoken language is accompanied by some form of signed communication. Sign-supported speech (SSS), also known as ‘simultaneous communication,’ is a form of TC in which the signed lexicon accompanying spoken language is borrowed from the indigenous sign language, sign markers are minimally used and not every single word is necessarily signed. As far as possible, signs and spoken language are produced simultaneously following the syntax of the spoken language (Spencer, 2016). This is different from natural code-blends produced by bimodal bilinguals, in which either spoken or sign language can be the matrix language that provides the syntactic structure of the sentence (Emmorey et al., 2015).

Arguments in favor of a purely OC approach with deaf children have stressed the importance of relying only on speech and hearing for communication in order to achieve the greatest auditory benefits from any sensory aid (Chan et al., 2000; Yoshida et al., 2008; Ingvalson and Wong, 2013). Proponents of a TC approach argue that some form of signs or gestures accompanying speech enhance language acquisition by providing deaf children with an additional source of information and continuous exposure to language (Knoors and Marschark, 2012). Findings of studies on the education of children with CIs are contradictory and have supported both the TC approach (McDonald Connor et al., 2000; Miyamoto et al., 2009) and the OC approach (Geers and Sedey, 2002; Geers et al., 2003). Consequently, it is unclear whether the adoption of a TC program would prevent children with CIs from the development of spoken language skills (Spencer et al., 2003; Spencer and Tomblin, 2006). When SSS is properly used, with naturally flowing spoken language, there is no evidence of a negative impact of the use of signs accompanying speech. Moreover, the information redundancy made available from the visual and auditory channels is thought to foster the acquisition of more complex material (Blom and Marschark, 2015). Studies that provide evidence for a positive effect of TC in children with CIs observed that this approach enhanced the acquisition of vocabulary, both spoken and signed. In particular, signed communication used before implantation has been found to be a useful bootstrap for spoken vocabulary acquisition (Yoshinaga-Itano, 2006). An advantage for oral expressive vocabulary acquisition, in children with CIs exposed to bimodal bilingualism (spoken and sign language) from an early age, was also found in a study with Spanish children (Jiménez et al., 2009). The authors reported that the bilingual children were able to evoke a greater number of words using pictures as stimuli, and showed greater verbal fluency, compared to children only exposed to OC programs. Furthermore, it has been found that children who received the CIs before the age of 5 years benefitted more than other deaf children in receptive spoken vocabulary scores from the exposure to TC settings compared to OC settings (McDonald Connor et al., 2000). These latter findings address a relation between age of implantation and communicative mode.

However, Giezen (2011) and Giezen et al. (2014) pointed out that most studies on deaf education, aside from case studies (De Raeve et al., 2009), have compared achievements of children exposed to OC with achievements of children exposed to TC or bilingual educational settings. To improve reliability, Giezen et al. (2014) advocated a comparison of these two educational settings by planning a within-subject design that tested whether the same deaf children benefitted more from a TC or OC setting. Their goal was to explore whether the use of signs at the same time as spoken words enhanced comprehension in deaf children, or if the two sources of information competed and reduced comprehension, compared to when perceiving spoken language-only. They compared the effects of sign language, spoken language and SSS in word processing in a small group of children wearing CIs. The children showed greater accuracy and faster reaction times in the SSS than in the spoken language condition, suggesting that the bimodal condition might involve a cross-modal facilitation in lexical processing. In line with previous studies (McDonald Connor et al., 2000; Knoors and Marschark, 2012), their results suggested that SSS would be particularly beneficial with deaf children with early implantation and relatively strong spoken language proficiency. Whether SSS could be more effective than spoken language also with children with no CIs, in particular with children with a native knowledge of sign language, should be investigated in more depth. Furthermore, Giezen et al. (2014) only addressed word-level processing, but stressed the relevance of extending this research to sentence and discourse levels to establish the effect of signed communication on spoken language in more demanding cognitive tasks.

The effectiveness of SSS might also depend on the content of the message. Prior literature has been interested in the particular suitability of sign language to communicate spatial contents (Emmorey et al., 2000), using space itself to transmit spatial information. Whether signed communication, sign language and SSS are more effective than spoken language-only in transmitting spatial contents was also addressed in the current study.

The potential strength of SSS with respect to spoken language is the additional visual channel of information of signs. How deaf individuals allocate their eye gaze while visually perceiving SSS might also give us information about the relevance of multiple sources of linguistic cues for deaf perceivers. This was done in the current study by recording eye movements.

Results from research on gaze behavior during language perception show that there is a tendency to look at a speaker’s face during face-to-face communication (Argyle and Cook, 1976). This has been attributed to strong social norms which produce an “eye primacy effect” (Gullberg and Holmqvist, 1999; Lansing and McConckie, 1999). This gaze behavior has also been reported in deaf people perceiving sign language (Siple, 1978; Muir and Richardson, 2002; Agrafiotis et al., 2003; Emmorey et al., 2009), and is thought to be because large moving targets, such as signs in sign languages, are successfully perceived through peripheral vision (Swisher et al., 1989). The visual area from which useful information can be obtained, including central foveal vision (i.e., what a fixation measure would register) and peripheral vision, is referred to as the “useful visual field” (Saida and Ikeda, 1979). When signs are articulated far away from the face area, the useful visual field might not include face and signs at the same time, so that more foveal fixations toward the signs might occur. This is just what was found in an eye-tracking study on gestures accompanying speech (Gullberg and Holmqvist, 1999).

The area of the face attended during sign language perception has also been debated. Level of expertise in sign language might dictate perceivers’ gaze, with people learning to sign looking more at the mouth area and native signers looking more at the eyes area (Emmorey et al., 2009). The authors hypothesized that beginning signers looked more extensively at the mouth in order to pick up the additional information provided by mouthings, which may not be necessary for native signers. In fact, almost all sign languages present specific mouth patterns associated to signs and time locked to the signs’ manual component articulation (Boyes Braem and Sutton-Spence, 2001; Sutton-Spence, 2007). Moreover, stimuli presentation of signed communication, through live interaction or video-recordings, also affect perceivers’ gaze. Deaf perceivers have been shown to focus primarily on the eye area during live interactions (Emmorey et al., 2009), but on the mouth area while watching video-clips of a signer (Muir and Richardson, 2002; Agrafiotis et al., 2003). Despite these differences, a general bias in gaze fixations toward the face area compared to the body area was reported by all these studies, providing evidence for the feasibility of collecting reliable eye movement data of signed communication perception in live interactions as well as while watching video-clips.

The relevance of peripheral vision in deaf individuals has also been demonstrated in studies of reading skills (Bélanger et al., 2012) and visual selective attention tasks (Dye et al., 2009), which propose that peripheral vision is more developed in deaf than in hearing people. The use of peripheral vision in sign perception has also been explored during perception of SSS. De Filippo and Lansing (2006) presented basic sentences in SSS containing a pair of ‘sign-critical’ or ‘speech critical’ contrast items. Sign-critical contrasts included two homophones that are signed differently, therefore only the sign disambiguated the meaning of the sentence. Speech-critical contrasts included a sign that corresponds to two different words, therefore only the speech disambiguated the meaning. Eye movement data indicated that even when perceiving sign-critical contrasts participants looked primarily at the face, and they still achieved high accuracy in identifying the disambiguating sign. These findings contribute to evidence demonstrating that information is gained from the hands even when deaf individuals mostly look toward the face when perceiving SSS.

Additional information about eye gaze in deaf individuals can be obtained by a comparison between the distribution of fixations when attending spoken and signed languages and SSS, in order to shed further light on the gaze behavior of deaf perceivers when exposed to unimodal or bimodal linguistic input. However, the question of whether SSS aids understanding of language in the context of discourse perception remains unanswered. Ultimately, an increase in comprehension in SSS compared to OC, combined with eye fixations that remain primarily oriented toward the face area, would suggest that participants benefit from signed input peripherally perceived. The motivation for the current study was to address two main weaknesses in the literature on the effects of SSS in transmitting information: a lack of research in testing SSS at a discourse level, and a lack of studies examining comprehension within the same group of individuals. Specifically, our goal was to explore whether the dual input of information offered by SSS was more effective in transmitting information than the unimodal input of spoken language. Moreover, we intended to investigate whether SSS was particularly useful for communicating spatial information.

Three primary research questions were addressed. First, we examined whether deaf individuals comprehended SSS to the same level that hearing individuals comprehended spoken language. Second, we examined if SSS was better comprehended than spoken language and Spanish sign language (LSE) across (i) all deaf participants, (ii) prelingually profoundly deaf CI users, and (iii) profoundly deaf native LSE signers. Third, we investigated whether SSS was more effective than spoken language in transmitting spatial information compared to non-spatial information by analysing comprehension scores. We also examined whether comprehension achievements correlated with linguistic and cognitive skills: lip-reading, spoken receptive vocabulary size, proficiency in spoken and signed language, non-verbal IQ and working memory.

Further research questions aimed to broaden our understanding of the mechanisms that govern gaze behavior during language perception in deaf individuals. To this purpose, we first explored if eye behaviors of deaf individuals differed from hearing participants while perceiving spoken language. Second, we examined if the distribution of gaze fixations in the deaf group varied depending on the channel of the linguistic input, spoken, signed or spoken + signed. In particular, we addressed the question of whether the gaze behavior differed on the basis of access to the native knowledge of LSE (focusing on native LSE signers). Third, we tested if deaf participants looked more extensively toward the signs when details of spatial contents were provided. We also examined whether native LSE signers and non-native LSE users differed in spoken language, LSE proficiency and lip-reading skills, and how these possible differences related to gaze behavior during LSE and SSS perception.

With respect to the effectiveness of SSS in enhancing comprehension, we expected that deaf participants would achieve the same comprehension in SSS as hearing participants in spoken language. Namely, SSS could bootstrap comprehension more than spoken language in CI users, who are supposed to profitably rely on both sign and auditory inputs (Knoors and Marschark, 2012). We also expected that native LSE signers would have a better understanding of SSS than spoken language, because the support offered by the signs would be more beneficial than the support offered by lip movements, which can be only partially discerned even by expert lipreaders (Bernstein et al., 2000; Kelly and Barac-Cikoja, 2007). However, although native LSE signers might benefit from SSS more than spoken language-only, they are likely to achieve the highest level of comprehension when attending the message in their native language.

Regarding eye movements, deaf participants were expected to allocate their eye gaze more around the mouth than the eyes compared to hearing participants, when perceiving spoken language, in order facilitate lip-reading (Andersson et al., 2001). Deaf participants were expected to look at the face area more than the signs when perceiving LSE and SSS (De Filippo and Lansing, 2006; Emmorey et al., 2009). Native LSE signers were hypothesized to look at the face area to a larger extent than later signers, being more likely to have developed an ability to perceive signs through peripheral vision (Agrafiotis et al., 2003). Regarding the perception of the face, looking at the lower part of the face rather than at the upper part might be preferable when perceiving LSE and SSS (Muir and Richardson, 2002; Agrafiotis et al., 2003) in order to visualize larger portions of the sign model through peripheral vision. A bias for fixating on the lower area of the face more than the upper area might be especially evident in non-native LSE users (Emmorey et al., 2009), since non-native LSE users are expected to rely on mouthings in LSE and lip-reading and speech in SSS more than native LSE signers.

However, since SSS follows the syntactic structure of spoken language, the unnatural order in which signs appear might drive native LSE signers gaze toward the unexpected information from signs.

Lastly, we expected more fixations toward the signs conveying spatial information due to the spatial nature of the signs themselves, and due to the fact that directional signs were in general articulated in a more peripheral area of the signing space, which might affect the gaze behavior (Gullberg and Holmqvist, 1999).

## Materials and Methods

### Participants

The final sample was composed of 25 deaf students (12 male, 13 female; mean age = 14.97 years; *SD* = 0.37) and 25 hearing students, matched on chronological age with the deaf group (13 males, 12 females; mean age = 14.91 years; *SD* = 0.37). Data from five additional deaf students was excluded because their non-verbal IQ was below 70 and they were unable to meet criteria on an N-back working memory task. Participants were recruited from mainstream schools or special education institutions for deaf individuals in the areas of Andalusia and Madrid, in Spain.

Individual background information for deaf participants, including spoken language and LSE proficiency and lip-reading proficiency, is provided in **Table 1**. Background skills – non-verbal IQ, working memory, spoken receptive vocabulary size – for deaf and hearing participants are provided in **Table 2**. All deaf participants had at least basic skills in LSE, acquired in family, school or speech and language therapy contexts. Three students (participants 4, 23, and 25 in **Table 1**) had recently learnt LSE by using it with other deaf students and with LSE/Spanish interpreters at school. These students, who all had a severe degree of deafness, were included in a preliminary analysis comparing discourse comprehension in hearing participants and deaf participants as a whole since the lexical items used in the experimental tasks were high frequency and should be known even by beginner LSE users. Other participants who achieved low scores in language proficiency, despite several years of LSE use, were included in the analyses for the same reason.

**Table 1:** Background characteristics of deaf participants.

Participant	Stimulation	Age of implantation in years	Parents’ language	Degree of hearing loss	Age of hearing loss in years	% Lip-reading	% Spoken language proficiency	% LSE proficiency
1^a^	CI	>3	Spanish	P	>3	–	90	40
2	HA		Spanish	S	≤3	39	80	70
3	HA		LSE	MS	≤3	73	90	80
4^b^	HA		Spanish	S	≤3	45	70	20
5^c^	CI	≤3	LSE	P	Birth	64	80	90
6	HA		LSE	P	Birth	26	60	100
7	CI	>3	Spanish	P	>3	2	100	50
8	HA		Spanish	S	≤3	17	100	50
9	HA		Spanish	S	>3	61	80	70
10	CI	≤3	Spanish	P	Birth	61	100	70
11	CI	≤3	Spanish	P	Birth	81	60	80
12	CI	≤3	Spanish	P	≤3	36	90	90
13	HA		LSE	P	Birth	30	40	60
14^a^	HA		LSE	P	Birth	–	–	90
15	HA		Spanish	MS	Birth	46	100	80
16	HA		Spanish	MS	≤3	39	80	60
17	HA		LSE	P	Birth	4	30	100
18	CI	≤3	Spanish	P	≤3	54	90	80
19	HA		LSE	P	Birth	4	80	100
20	CI	≤3	Spanish	P	Birth	51	100	80
21	CI	>3	Spanish	S	Birth	25	80	80
22	HA		Spanish	P	>3	24	50	20
23^a,b^	HA		Spanish	S	Birth	78	80	–
24^d^	HA		Spanish	P	Birth	11	70	70
25^b^	HA		Spanish	S	Birth	40	100	40


*CI, cochlear implants; HA, hearing aids; P, profound deafness; S, severe deafness; MS, moderate to severe deafness. Lip-reading was measured by Spanish adaptation of Utley test (Manrique and Huarte, 2002); Spanish spoken language proficiency and LSE proficiency were assessed by using non-standardized tasks developed by Rodríguez-Ortiz (2005). ^a^These participants did not complete one or more of the tests related to spoken language and LSE proficiency or lip-reading, due to difficulties with arranging further testing sessions. ^b^Beginning signers not included in the gaze behavior analyses comparing native LSE signers and non-native LSE users. ^c^Participant, native signer and CI user, excluded from subgroup analyses. ^d^Participant, with a high level of missing eye tracking data, excluded from gaze behavior analyses.*

**Table 2:** Total scores on cognitive skills and spoken receptive vocabulary size across deaf and hearing groups.

GROUP	Non-verbal IQ score	WM 1-back % accuracy	WM 2-back % accuracy	WM 3-back % accuracy	Spoken receptive vocabulary
Deaf group (*n* = 25)	99.96 (10.03)	87.56 (9.78)	77.76 (13.54)	62.60 (18.39)	67.32 (22.75)
Hearing group (*n* = 25)	101.98 (10.44)	92.96 (7.98)	83.56 (16.62)	74.44 (18.36)	106.36 (18.49)


*Mean scores in the background skills (standard deviations are in parentheses). Non-verbal IQ was measured by Raven’s Standard Progressive Matrices test (Raven et al., 1995); working memory (WM) was measure by the n-back test (Kirchner, 1958); spoken receptive vocabulary was measured by Spanish Peabody Picture Vocabulary test (Dunn et al., 2006).*

All deaf participants had at least some exposure to SSS at school. Participants mainly experienced forms of SSS outside of the main classroom, for example when reviewing specific subjects in resource rooms, with other deaf students and the itinerant teacher of the deaf, and/or during speech therapy. Some participants attending an educational setting with co-enrolment teaching were likely to be exposed to SSS to a greater extent.

Kolmogorov–Smirnov test was used to test the data distribution across hearing and deaf participants in cognitive skills and receptive vocabulary. The distribution for non-verbal IQ was equivalent across deaf (*M* = 99.96, *SD* = 10.03) and hearing participants (*M* = 101.98, *SD* = 10.44), *K-S Z* = 0.57, *p* = 0.91. The N-back working memory task was tested across three progressive levels of difficulty. Data distribution was equivalent at 1-back level (deaf: *M* = 87.56, *SD* = 9.78; hearing: *M* = 92.96, *SD* = 7.98; *K-S Z* = 1.13, *p* = 0.16), but the hearing group scored higher than the deaf group on the 2-back (deaf: *M* = 77.76, *SD* = 13.54; hearing: *M* = 83.56, *SD* = 16.62; *K-S Z* = 1.41, *p* < 0.05), and the 3-back level (deaf group: *M* = 62.60, *SD* = 18.39; hearing group: *M* = 74.44, *SD* = 18.36; *K-S Z* = 1.697, *p* < 0.01). As expected, a significant difference between deaf and hearing participants was detected on spoken language receptive vocabulary size (deaf: *M* = 67.32, *SD* = 22.75; hearing: *M* = 106.36, *SD* = 18.49; *K-S Z* = 2.55, *p* < 0.001).

Participants’ deafness-related characteristics were as follows:

- LSE signing: 7 participants were native LSE signers, 15 participants were LSE high-proficient users, 3 participants were LSE basic users.
- Age of hearing loss: 19 participants were deaf from birth or before age 2, 6 participants were diagnosed as deaf after or at age 3.
- Degree of hearing loss: 9 participants were moderately to severely deaf, 16 participants were profoundly deaf.
- Use of CI: 9 participants used unilateral CIs, 16 participants used uni- or bilateral hearing aids.

Within this heterogeneous group of deaf participants, we identified more cohesive subgroups for discourse comprehension contrasts: five participants with a diagnosis of profound hearing loss before 2 years of age and with CIs before 5 years old; five profoundly deaf participants with deaf parents, thus with native LSE knowledge. One participant was left out of subgroup analyses because she shared characteristics of both subgroups: she was a native LSE signer and had early CIs (participant 5).

For the eye tracking analyses to test the effects of native competence in LSE on the distribution of gaze fixations, deaf participants were grouped on the basis of their native (*N* = 6) or non-native knowledge of LSE (*N* = 14), regardless of the degree of hearing loss. Overall, five deaf participants were left out of eye movements’ analyses: the native LSE signer who wore early CIs, a participant with a high level of missing eye tracking data, and the three beginner LSE users.

### Instruments and Tasks

#### Measures for Cognitive and Linguistic Skills

All participants completed the following cognitive and linguistic measures in a randomized order.

- Raven’s Standard Progressive Matrices (Raven et al., 1995). This is a well-established non-verbal test assessing general cognitive ability. The Standard Progressive Matrices version consists of 60 non-verbal multiple choice questions grouped in five sets of increasing difficulty. Each item requires the participant to identify the missing element between six or eight options to complete a pattern.
- N-back test (Kirchner, 1958). This task assesses working memory capacity and is frequently used in cognitive neuroscience research. The n-back task adopted here was programmed by Robinson and Fuller (2004, unpublished) in E-prime (Schneider et al., 2002). This is a computerized continuous recognition task in which participants must decide whether a stimulus is the same as the one presented “*n*” items back. Responses are made by pressing ‘yes’ or ‘no’ on a keyboard. The stimuli consist of letters and there are three levels of working memory load (1-back, 2-back, and 3-back). At the 1-back level, participants compare the letter currently displayed on the screen with the one presented immediately before, at the 2-back level, with the letter shown two positions prior, and at the 3-back level, with the letter that appeared three positions prior. Higher executive control processes are involved in the 2-back and 3-back conditions of the task compared to the 1-back condition (Pelegrina et al., 2015).
- Test de Vocabulario en Imágenes de Peabody [Spanish Peabody Picture Vocabulary Test – TVIP] (Dunn et al., 2006). This measure assesses receptive vocabulary in Spanish. It consists of a list of words clustered in different sets of increasing difficulty, corresponding to the predicted receptive vocabulary knowledge at different age ranges. Participants are required to select the picture depicting the meaning of each word from four possible images.

Deaf participants were also assessed in the comprehension of Spanish spoken language and LSE and in lip-reading skills.

For assessing comprehension of Spanish spoken language and LSE, a text (500 words long) for each communicative mode was recorded and shown to participants. Comprehension was assessed through a set of ten true or false questions. In order to avoid an excessive working memory load, the texts were administered in three blocks along with the respective questions (see Rodríguez-Ortiz, 2005, for additional information on this task). Scores from these measures of spoken language and LSE are referred to as spoken language proficiency and LSE proficiency in the following sections and are correlated with the scores obtained in the comprehension of the experimental tasks in the three communicative modes: SSS, spoken language and LSE.

For assessing lip-reading skills, the Utley test was used (Utley, 1946, adapted in Spanish language by Manrique and Huarte, 2002). This measure assesses lip-reading skills of participants. We used the form A of Utley test consisting of 31 sentences of up to 5 words, and a total of 106 words. Participants are required to watch each video once without sound and are then asked to repeat the sentence aloud, or to write it down in case their language articulation was not clear.

#### Stimuli

Six stories were presented by a bimodal bilingual Spanish-LSE native user, a hearing woman with deaf parents, previously trained to the use of SSS and supervised during stimuli video-recording. Stimuli were recorded in three communicative modes: SSS, spoken language and LSE and presented with sound. Two stories were shown in each communicative mode. The texts had a mean length of 85 s (*min* = 73, *max* = 94) when recorded in spoken language, of 109 s (*min* = 104, *max* = 113) when recorded in SSS, of 106 s (*min* = 99, *max* = 113) in LSE. Video-clips in SSS and in LSE are usually longer than video-clips where the same information is expressed in spoken language because the use of signs tends to produce utterances of longer duration (Whitehead et al., 1997). However, signed messages were produced aiming to maintain the stream of discourse as naturally as possible, as advocated in Knoors and Marschark (2012). Mouthings accompanied about 64% of the signs in LSE stories, marginally less than the amount of mouthings counted for the British Sign Language database (69%; Sutton-Spence, 2007). The lexical items used in these stories were mostly high frequency in spoken language, as reported in a Spanish lexical database (CREA–Corpus Real Academia Española^1^). Testing materials included the description of spatial relationships. In SSS, spatial information was communicated by using the citation forms of LSE cardinal signs and signs for lexical relational terms left/right and using the space to locate the referents in their mutual position. Below are two examples of how SSS was implemented (in English translation), in particular in the transmission of spatial contents. By convention, the signs are transcribed in capital letters as spoken language glosses.

(1) ‘Their house was between the house of a fisherman, on the left, and a park on the right.’
  HOUSE BE HOUSE FISHERMAN LEFT PARK RIGHT
(2) ‘The cat ran away toward the north and turns to the right, hiding itself in a kiosk in the corner.’
  CAT RUN NORTH TURN RIGHT HIDE KIOSK CORNER

**FIGURE 1:**
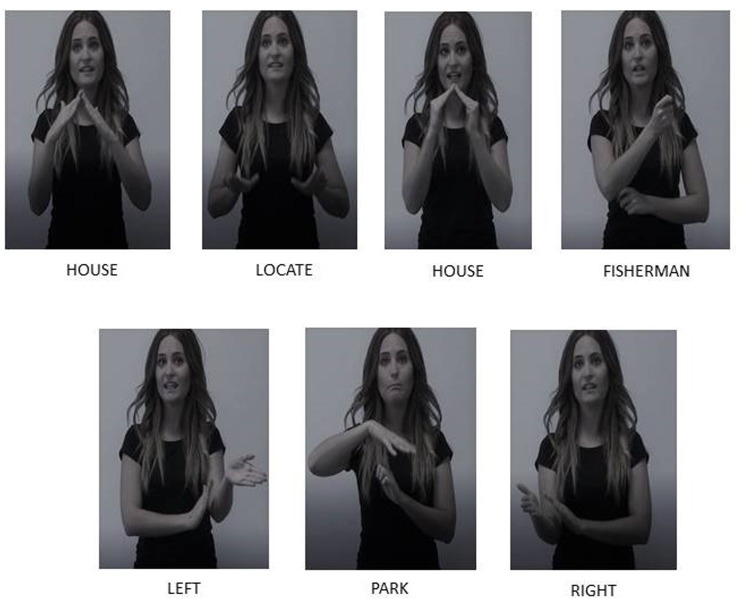
Example of the use of SSS in transmitting spatial information. The signs in the figure accompanied the Spanish spoken sentence “Sus casa estaba entre la casa del pescador a la izquierda y un parque a la derecha” (“Their house was between the house of a fisherman, on the left, and a park, on the right”).

The signs used in the SSS sentence example (1) are also shown in **Figure 1** and are included in the story provided in Supplementary Table S1. The SSS sentence example (2) includes the motion verb TURN followed by the directional sign RIGHT. In this example, the sign TURN moves toward the right side of the sign model and the next sign for CORNER is shown as right-sided corner, as well as the sign for KIOSK, the endpoint of the spatial description. This redundancy in communicating spatial information provided by signs in SSS is not present in spoken language-only. In the spoken modality, spatial information is communicated by only using the relational term “right.” The reinforced spatial message transmitted through SSS might facilitate the information uptake. Motion verbs were frequent in these texts where a route perspective was frequently adopted instead of a survey perspective. The route perspective uses a viewer spatial format where the viewer is conceived as immersed in the described environment (Emmorey et al., 2000).

Short stories were used rather than basic sentences because discourse comprehension of SSS has not been thoroughly explored. The maximum score achievable in each story was 8, therefore a maximum of 16 correct answers was obtainable in each communicative mode (SSS, spoken language, LSE). Comprehension was assessed through three multiple-choice questions and the identification of five locations on a map. Instructions and a practice trial were given in SSS. An example of the texts and tasks is shown in Supplementary Table S1.

### Apparatus

Stimuli were presented on a 15.6-inch ASUS monitor at a resolution of 1366 × 768 pixels and a refresh rate of 60 Hz. Participants’ eye movements during discourse comprehension was tracked using an EyeLink 1000 eye tracker with a head-chin rest system (SR Research, Ottawa, ON, Canada). The head-chin rest ensured a viewing distance of 60 cm. The size of the model on the screen was 510 × 470 pixels with visual angle of 11° in width and 17° in height. The camera mount used was desktop mount with camera level orientation and a monocular eye tracking, with illuminator on the right. A 35 mm lens was used and a thirteen-point calibration type for recording was selected for most participants, allowing a high degree of accuracy. Three participants wore prescription glasses so a nine-point calibration was used. The sampling rate for recording was 1000 Hz.

### Procedure

The experiment was programmed in SR Research Experiment Builder (SR Research, Ottawa, ON, Canada). Three lists of texts were created. Each list included each text only in one of the three communicative conditions and was administered to a third of the sample. In total, participants were required to watch six videos in a randomized order: three descriptive and three narrative texts, two for each condition (SSS, spoken language and LSE). A practice video recorded in SSS was viewed before the experimental trials. Each story was shown twice; after the first viewing participants were asked to complete the multiple choice questions, and after the next viewing participants had to complete the map task. The task was presented with written questions, but participants could also watch the questions video-recorded in SSS if they had difficulties understanding the written form. The test was administered in one session of approximately 1 h in length, including breaks. No time limit was set. After each video-clip a blank screen with a central fixation point was shown and participants could take a break. Re-calibration was completed before continuing if necessary. A manual command had to be given before continuing to the next trial. Instructions, presented using Spanish SSS and displayed with Spanish subtitles, are translated as follows: “*Hi, we are going to watch short video-clips in which a person describes some stories or places using either spoken language-only, LSE-only, or spoken language accompanied by signs. At the end of each video-clip you will be asked to answer some questions about the story. Then, we will watch the same video again, along with a map. You should pay attention to the places described and find them on the map. Now we will watch a trial video-clip: look at it carefully and answer the questions*.”

Ethical standards were in compliant with Declaration of Helsinki principles and all subjects or their legal tutors if minors gave written informed consent. Ethical approval was obtained from the Andalusian Committee for Biomedical Research.

### Data Preparation

The EyeLink gaze data were viewed, filtered and processed using the SR Research Data Viewer (SR research, Ottawa, ON, Canada). Tab-delimited data files were exported for the analyses, which were carried out in IBM SPSS 21.

**FIGURE 2:**
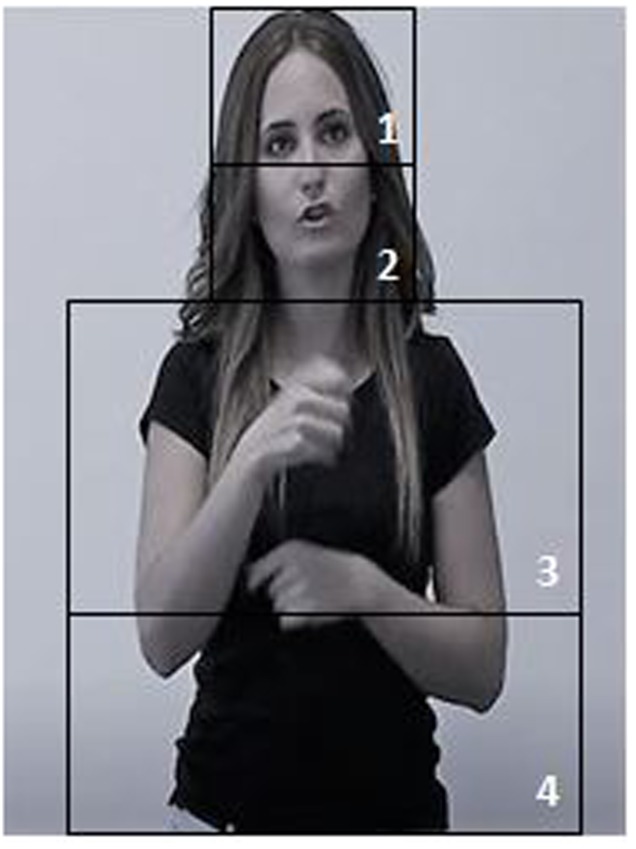
Interest areas on the signer’s body: 1 = Upper face; 2 = Lower face; 3 = Upper body; 4 = Lower body.

A set of static interest areas (IAs) with a rectangular shape were created on the sign model figure: the “upper face” (including the area around the eyes, the forehead and the upper part of the nose), the “lower face” (including the area around the mouth, the chin and upper part of the neck), the “upper body” (including shoulder and thorax) and the “lower body” (including the lower abdominal area), as shown in **Figure 2**. Given the very scant number of fixations toward the lower body, inspections in the upper and lower body were compiled in the analyses. Due to the reduced size of the figure in the video-clips, finer-grained areas were not defined. Report variables relating to dwell times (DTs) in the raw data were inspected; ‘total DT’ indicates how much time was spent in an IA over the whole trial (Holmqvist et al., 2011). Eye tracking data from all trials of the whole sample were included in the analysis, with the exception of one deaf participant who was excluded due to large amounts of missing data. Duration-based interest periods were also set, based on the start/end timings of each of the five “location” events for each trial. To analyze gaze behavior when perceiving critical sentences, eye-gaze during critical sentences that included spatial information was compared to eye-gaze during non-critical sentences that included any non-spatial information.

## Results

### Analytic Approach

The analyses aimed to examine the benefits of using the dual input of sign and speech jointly (SSS) compared to the unimodal input of spoken language-alone and LSE-alone in deaf participants.

To this aim, we first compared the scores in each communicative system (SSS, spoken language, LSE) of the deaf group, including all 25 participants, with spoken language comprehension of the hearing group.

Next, we focused solely on the group of deaf students. Comprehension performance (percentage accuracy) was compared across the three communicative systems for the whole deaf group. We then examined comprehension performance in the homogeneous subgroup of prelingually profoundly deaf early CI users (*n* = 5) and in the homogeneous subgroup of profoundly deaf native LSE signers (*n* = 5). This was done first for all questions, and then for the map task questions that transmitted spatial information. Finally, we correlated scores on linguistic and cognitive tasks with comprehension performance.

With respect to the gaze behavior, we first compared eye movements of deaf participants and hearing participants when perceiving spoken language. We analyzed the DTs (in seconds) spent fixating different areas of the sign model, body and face areas, and in more detail, upper and lower areas of the face. Next, we compared the distributions of fixations within the deaf group across the three communicative systems. We examined whether severely and profoundly deaf participants, native signers (*n* = 6) and non-native LSE users (*n* = 14), differed with respect to DTs on the face and body during SSS and LSE communication. We also compared native and non-native LSE users in spoken language proficiency and LSE proficiency and lip-reading skills (**Table 3**), and tested if possible differences were reflected in gaze behavior during LSE and SSS perception. Finally, we explored gaze behavior of deaf participants and specifically native LSE signers when perceiving spatial information in LSE and SSS, testing our expectations that higher DTs were registered in the body area, where most of the signs were performed, when spatial information was transmitted. We took into account the interest periods in which critical information relating to a spatial description was transmitted. The percentage of total DT when participants gazed at the body IA during critical sentences with respect to the total duration of critical sentences was compared to the percentage of DT on the body IA when no spatial information was communicated.

**Table 3:** Total scores on linguistic skills across native profoundly and severely deaf native LSE signers and non-native long-term LSE users.

GROUP	% LSE proficiency	% Spoken language proficiency	% Lip-reading
Native LSE signers (*N* = 6)	88.30 (16.02)	60.00 (25.50)^a^	27.40 (28.21)^a^
Non-native LSE users (*N* = 14)	65.70 (19.50)	85.70 (15.50)	41.23 (21.15)^a^


*Mean scores in linguistic skills (standard deviations are in parentheses). Lip-reading was measured by Spanish adaptation of Utley test (Manrique and Huarte, 2002); Spanish spoken language proficiency and LSE proficiency were assessed by using a non-standardized task developed by Rodríguez-Ortiz (2005). ^a^Data reported for five participants.*

When data were non-normally distributed, before using non-parametric statistics—Mann–Whitney *U* test across groups and Friedman and Wilcoxon Signed Rank test across conditions within groups— we verified whether distribution could be normalized by log_10_ transformation. For correlation analyses, Pearson product-moment correlation coefficient was computed. For parametric contrasts, Cohen’s *d* is reported as an effect size measure. For non-parametric analyses, correlation effect size *r* was considered to be more appropriate (Fritz et al., 2012). Effect sizes can be considered large for *d* values higher than 0.8 and *r* values higher than 0.37, intermediate from 0.5 and 0.24, and small for values lower than 0.2 and 0.10, respectively. The adjusted degrees of freedom were reported when data violated the assumption of equal variance between groups.

### Analysis of Discourse Comprehension

The hearing group (*n* = 25) achieved significantly higher scores in the comprehension of spoken language (*M* = 0.83, *SD* = 0.12) than the deaf group (*n* = 25) in the comprehension of any communicative system used, [SSS (*M* = 0.58, *SD* = 0.20), *t*(39.12) = -5.17, *p* < 0.001, *d* = 1.43; spoken language (*M* = 0.54, *SD* = 0.26), *t*(33.69) = -5.00, *p* < 0.001, *d* = 1.52, and LSE (*M* = 0.50, *SD* = 0.27), *t*(33.06) = -5.52, *p* < 0.001, *d* = 1.58].

Within the deaf group, non-parametric Friedman test revealed that the effect of the communicative system (SSS, spoken language or LSE) on comprehension was non-significant, Chi-Square = 1.195, *p* = 0.550.

When considering the subgroup of deaf participants that were early CI users (*n* = 5), their comprehension of SSS (*M* = 0.66, *SD* = 0.24) did not differ from comprehension of hearing participants, *t*(4.42) = -1.52, *p* = 0.20, *d* = 0.89. Similarly, early CI users did not differ from hearing participants when comprehending spoken language (*M* = 0.51, *SD* = 0.31), *t*(4.24) = -2.23, *p* = 0.08, *d* = 1.36, or LSE (*M* = 0.66, *SD* = 0.24), *t*(4.20) = -1.78, *p* = 0.14, *d* = 1.10. Comprehension in early CI users did not differ across the three communicative systems, SSS, spoken language and LSE, Chi square = 2.53, *p* = 0.28.

When considering only profoundly deaf native LSE signers (*n* = 5), analysis revealed that they reached a comparable level of comprehension to the hearing group when discourse was transmitted in their native language, LSE (*M* = 0.73, *SD* = 0.22), *t*(4.47) = -0.99, *p* = 0.37, *d* = 0.56. Conversely, they achieved lower scores than the hearing peers when evaluated in spoken language (*M* = 0.41, *SD* = 0.06), *t*(28) = -7.49, *p* < 0.001, *d* = 4.43, as well as in SSS (*M* = 0.49, *SD* = 0.21), *t*(28) = -5.05, *p* < 0.001, *d* = 1.99. Within group analyses demonstrated no significant differences in level of comprehension between SSS and spoken language, *Z* = -0.96, *p* = 0.34, *r* = 0.25, whilst LSE was comprehended more successfully than spoken language, *Z* = -2.02, *p* < 0.05, *r* = 0.70.

The only profoundly deaf native LSE signer with CIs, who was excluded from the subgroup analyses, achieved the same level of comprehension in LSE (75%) as the other native LSE signers and she achieved higher comprehension scores in spoken language and SSS (94%) – more than one standard deviation above profoundly deaf native LSE signers. This participant also scored more than one standard deviation above early CI users in SSS and spoken language and her scores in spoken language were similar to those of hearing controls.

To determine whether a signed communication (SSS and LSE) was more effective than spoken language in transmitting spatial information we compared the percentage of scores achieved in the map task across the three communicative systems. Analyses including all deaf participants did not reveal significant differences in comprehension of spoken language (*M* = 0.51, *SD* = 0.33) compared to SSS (*M* = 0.56, *SD* = 0.25), *Z* = -0.69, *p* = 0.49, *r* = 0.14, or to LSE (*M* = 0.46, *SD* = 0.31), *Z* = -0.41, *p* = 0.68, *r* = 0.08. The early CI users also achieved comparable comprehension between spoken language (*M* = 0.50, *SD* = 0.46) and SSS (*M* = 0.66, *SD* = 0.30), *Z* = -1.13, *p* = 0.26, *r* = 0.20, and spoken language and LSE (*M* = 0.52, *SD* = 0.37), *Z* = -0.13, *p* = 0.89, *r* = 0.02. Similarly, profoundly deaf native LSE signers scored equally in spoken language (*M* = 0.32, *SD* = 0.16) compared to SSS (*M* = 0.44, *SD* = 0.27), *Z* = -0.96, *p* = 0.34, *r* = 0.26, and compared to LSE (*M* = 0.60, *SD* = 0.41), *Z* = -1.21, *p* = 0.22, *r* = 0.39.

Finally, correlations between linguistic/cognitive skills and comprehension in deaf participants were analyzed. We only report correlations with a relationship of medium or large strength. Regarding lip-reading (Utley test), there was no correlation with accuracy either in SSS, *r*(23) = 0.36, *p* = 0.09, spoken language, *r*(23) = 0.31, *p* = 0.15, nor in LSE, *r*(23) = -0.36, *p* = 0.09. Regarding LSE proficiency, no correlation was found with accuracy in SSS, *r*(22) = 0.33, *p* = 0.13 but a significant large correlation was found with accuracy in LSE, *r*(22) = 0.54, *p* < 0.01. Regarding spoken language proficiency, a significant medium positive correlation was found with the experimental task on spoken language comprehension, *r*(24) = 0.42, *p* < 0.05. Regarding spoken receptive vocabulary (TVIP), no correlation was detected with accuracy in SSS, *r*(25) = 0.33, *p* = 0.10, but there was a significant correlation with accuracy in spoken language, *r*(25) = 0.48, *p* < 0.05. Working memory (N-back task) was not correlated at the 2-back level with accuracy in LSE comprehension, *r*(25) = 0.31, *p* = 0.13, but it was correlated at the 3-back level with accuracy in spoken language, *r*(25) = 0.48, *p* < 0.05. Non-verbal intelligence (Raven’s) correlated with LSE comprehension, *r*(25) = 0.56, *p* < 0.01.

### Analysis of Gaze Behavior

Both participant groups looked at the face significantly more than the body area when perceiving spoken language (hearing: 97.55%; deaf: 97.54%; both *p*’s < 0.001), and there were no significant group differences, (hearing: *M* = 70.33, *SD* = 11.82; deaf: *M* = 74.65, *SD* = 11.69), *U* = 217, *Z* = -1.66, *p* = 0.097, *r* = 0.23. Nevertheless, hearing and deaf participants differed with regards to the area of the face attended: deaf participants attended the lower face area 72.2% of total time spent on the whole face, for longer DTs (*M* = 54.27, *SD* = 23.83) than hearing participants (*M* = 36.62, *SD* = 24.18), who spent only 51.4% looking on the lower face, *U* = 166, *Z* = -2.68, *p* < 0.01, *r* = 0.38.

The deaf participants also looked at the face significantly more during SSS (95.83%) and LSE (95.93%). In the LSE condition, DT on the body was significantly less in the subgroup of native LSE signers (*M* = 0.42, *SD* = 0.33) than non-native LSE users (*M* = 5.11, *SD* = 7.19), *U* = 8, *Z* = -2.80, *p* < 0.01, *r* = -0.42. No differences were found in SSS condition (native signers: *M* = 5.98, *SD* = 7.00; non-native LSE users: *M* = 3.89, *SD* = 4.72; *U* = 40, *Z* = -0.16, *p* = 0.87, *r* = 0.17).

Then, we analyzed if differences in spoken language proficiency, LSE proficiency and lip-reading skills were reflected in gaze behavior, across severely and profoundly deaf native LSE signers and non-native LSE users. Means and standard deviations for these linguistic skills are reported in **Table 3**. Native LSE signers were significantly more proficient in LSE, *U* = 13.5, *Z* = -2.39, *p* < 0.05, *r* = 0.53, while non-native LSE users were more proficient in spoken language, *t*(17) = -2.68, *p* < 0.05, *d* = 1.17. Analyses did not reveal differences between groups in lip-reading scores, *t*(16) = -1.14, *p* = 0.27, *d* = 0.55, neither if comparing native LSE signers only to the subgroup of non-native LSE users, wearing CIs (*M* = 44.29, *SD* = 25.80), *t*(10) = -1.08, *p* = 0.31, *d* = 0.62. The lower proficiency of non-native LSE users in LSE did not reflect a greater attention for mouthings in LSE or lip-reading in SSS, rather both groups showed a similar preference for looking at the lower than the upper part of the face during LSE (native LSE signers: *M* = 72.52, *SD* = 34.96; non-native LSE users: *M* = 62.03, *SD* = 31.19; *t*(18) = 0.67, *p* = 0.51, *d* = 0.54), and during SSS perception, (native LSE signers: *M* = 81.47, *SD* = 25.01; non-native LSE users: *M* = 64.35, *SD* = 34.28; *U* = 31, *Z* = -0.91, *p* = 0.36, *r* = 0.27).

Given that native LSE signers and non-native LSE users did not differ in lip-reading skills, we computed a Pearson product-moment correlation coefficient for the deaf group as a whole, in order to test whether lip-reading abilities were related to the eye movements in language perception across participants. No correlation was found between lip-reading scores and DTs in the lower part of the face area while perceiving spoken language (*r* = -0.07, *n* = 22, *p* = 0.76), SSS (*r* = 0.29, *n* = 22, *p* = 0.20) or LSE (*r* = 0.41, *n* = 22, *p* = 0.06).

Finally, we investigated the effects of transmitting spatial information by signs on the gaze behavior of the whole deaf group and, more specifically, native LSE signers. As expected, the percentage of DT spent on the body IA was significantly higher when perceiving spatial information than during perception of other type of information. In the SSS condition, deaf participants gazed at the body area significantly longer when perceiving spatial information (*M* = 7.35, *SD* = 8.03) than when perceiving non-spatial information (*M = 5.59*, *SD* = 6.19), *Z* = -2.69, *p* < 0.01, *r* = 0.55. Similarly, during LSE comprehension, the percentage of DTs in the body IA when perceiving spatial information (*M* = 10.58, *SD* = 15.50) was higher than when perceiving non-spatial information, (*M* = 5.78, *SD* = 9.56), *Z* = -3.57, *p* < 0.001, *r* = 0.71. Narrowing down the analyses to the native- and non-native LSE subgroups, in the LSE condition native LSE signers spent significantly less DT looking at the body IA (*M* = 1.33, *SD* = 1.27) than non-native LSE users (*M* = 12.56, *SD* = 18.04), *U* = 17, *Z* = -2.06, *p* < 0.05, *r* = 0.40. By contrast, in the SSS condition, native signers looked at the body IA (*M* = 9.83, *SD* = 10.97) a similar amount to non-native LSE users (*M* = 7.62, *SD* = 7.63), *U* = 39, *Z* = -0.25, *p* = 0.80, *r* = 0.12.

## Discussion

This study covered two main topics: first, we investigated whether the use of dual linguistic input of SSS facilitated comprehension in deaf participants compared to the use of the unimodal input of spoken language and of sign language (LSE), in the case of non-native signers, when transmitting discourse. Second, we aimed to deepen our insight into the mechanisms that govern eye gaze of deaf perceivers when attending linguistic input from different channels, by observing eye movements in a sample of adolescents. With regards to language comprehension, we explored whether deaf participants were able to achieve levels of discourse comprehension equivalent to their hearing peers, and which communicative system (SSS, spoken language or LSE) was a better predictor of a successful comprehension. We also examined performance of subgroups within the deaf participant group: those with prelingual profound deafness with CIs, and those who had a native knowledge of LSE. A number of studies have shown that even if deaf children with CIs succeed in obtaining higher educational achievements than children with hearing aids and analogous hearing loss, they often have disadvantages compared to hearing peers (Marschark et al., 2007; Archbold et al., 2008). In the current study, the group of deaf participants, as a whole, did not achieve the same level of comprehension as their hearing peers. However, these difficulties were no longer evident in the prelingually profoundly deaf participants with early CIs. Their performance was equivalent to hearing peers in all communicative modes, highlighting the benefits of receiving the CIs at an early age. On the other hand, profoundly deaf native LSE signers achieved equivalent comprehension to control participants (using spoken language) when perceiving LSE, but not when perceiving spoken language or SSS. This highlights the importance of a native language for native LSE signers to achieve satisfactory language comprehension.

Within the deaf group there were no remarkable advantages in comprehension of SSS compared to spoken language when the deaf group was considered as a whole, nor when the analyses were narrowed down to participants with early CIs or to native LSE signers only. Our results are in line with previous accounts (Spencer et al., 2003; Spencer and Tomblin, 2006), indicating that the use of signs accompanying speech did not have a negative impact on the comprehension of spoken language. However, we still did not find evidence for a superiority of the redundant information supplied by SSS compared to spoken language-only, as reported by Giezen et al. (2014).

The only native LSE signer wearing CIs, who achieved higher spoken language and SSS scores than other deaf participants, represents a special case. Our results contribute to previous literature relating to this small population of deaf native signers with CIs, and are in line with previous findings where achievements in spoken vocabulary acquisition (Rinaldi and Caselli, 2014) and in a range of linguistic measures (Davidson et al., 2014) were comparable to those found in hearing peers. There is substantial agreement in explaining the development of good language skills and higher cognitive skills of deaf population through two conditions: a reduced period of auditory deprivation and an early language exposure (Figueras et al., 2008; Kronenberger et al., 2013, 2014). Individuals who underwent cochlear implantation at an early stage and are exposed since birth to LSE, such as participant 5 in the current study, meet both conditions responsible for normal developmental trajectories. As such, so long as no comorbid disturbances associated to deafness are present, a typical level of comprehension can be expected across modes of communication.

The lack of support for a comprehension advantage in perceiving SSS may partially be attributed to some limitations in the current study. First, the deaf population is vastly heterogeneous and there are a great number of variables that should be considered when forming comparison groups within this special population. Even taking into account most of the variables, the outcomes are not highly predictable, neither in children using CIs since an early age (Pisoni et al., 2010). There are also difficulties involved in collecting reliable data from all subjects. As such, despite best efforts to recruit a large sample and accurately characterize individuals, there were only a small number of participants in the subgroup analyses. The heterogeneity within the deaf group and the small number of participants with early CIs might have prevented the detection of main effects from the use of SSS.

A second limitation of this study might be the material used for evaluating comprehension. As Blom and Marschark (2015) pointed out, SSS might be more effective for transmitting more complex material, but the texts of our study did not vary a great deal in complexity. The primary difference between the current study and prior research (Giezen et al., 2014) was that they compared SSS and spoken language in single-word comprehension whereas we compared them at discourse level. On one hand, the assessment of discourse comprehension rather than isolated words and sentences is desirable as it gives a closer view of language comprehension in more natural communicative interactions. However, discourse comprehension involves higher cognitive components than single-word processing, such as memory and attentional processes. It also requires higher-level comprehension processes, such as generating inferences and constructing situation models of what text is about. The greater cognitive demands of discourse comprehension might have diminished the potential benefits of SSS in enhancing comprehension. For future research, texts of diverse complexity and difficulty should be used in order to investigate with what material SSS might produce higher benefits. Memory load should be controlled by measuring the length of the sentences used as stimuli, or presenting them in equal sections. Engaging participants by varying the difficulty level of content and the cognitive effort required may lead to distinct inferential processes and reveal differences in comprehensibility of SSS compared to other modes of communication.

As a consequence of the inconclusive findings related to benefits of SSS compared to spoken language-only, and the factors, sometimes unpredictable, affecting the language development of deaf children, targeted interventions adapting the more fruitful communicative approach at different stages of language development are recommendable (Lederberg et al., 2013; Martin Pérez et al., 2014). Ideally, all children, with or without CIs, should have an early exposure to sign communication that they will be able to use as an additional resource in communication, varying and adapting the use of signs to individual communicative needs (Knoors and Marschark, 2012).

Our results on gaze behavior replicated findings of previous studies in that the region which deaf participants primarily attended when watching the sign model on a video-clip was the face (De Filippo and Lansing, 2006). In our study, across all communicative systems – SSS, spoken language and LSE – deaf participants looked at the face more than 95% of the time. In particular, fixations were directed more often to the lower area of the face, around the mouth, which is in line with other studies using video-clips (Agrafiotis et al., 2003; Muir and Richardson, 2005). By contrast, hearing perceivers primarily looked at the upper face, around the eyes area, when perceiving spoken language. The tendency of deaf perceivers to focus on the lower face across all communicative systems might support the hypothesis that deaf individuals use peripheral vision for perceiving signs. The sign model was visible on the video-clip from the hips upward. Therefore, by fixating on a more central point than the eyes, in addition to focusing on lip-reading, participants could include a larger portion of signs performed around the body in their visual field. On the basis of LSE expertise, we expected that non-native LSE users would look at the lower part of the face to a higher extent than native LSE signers to compensate for the reduced fluency in signs with information from mouthings in LSE and from lip-reading in SSS. However, this was not found possibly because the small size of the sign model on the screen prevented the detection of differences between groups. Furthermore, there was no reason for non-native LSE users to look longer at the mouth because, although they were less skilled in LSE proficiency than native LSE signers, they had the same proficiency in lip-reading. However, overall, participants barely took their fixation off the face, mostly perceiving signs peripherally.

A foveal attention toward the signs was more likely to occur during the perception of spatial language, as already found in Emmorey et al. (2009), plausibly due to the suitability of signs in describing this kind of information. The higher attention for the signs did not result in a better comprehension of SSS compared to spoken language when spatial information was transmitted. It is possible that the map task which we used to test comprehension of spatial information was such a demanding memory task itself that it rendered the eventual advantages of SSS insignificant.

The use of peripheral vision in perceiving signs was even clearer in native LSE signers, who had less gaze deviations than other deaf participants toward the body during perception of LSE. This gaze behavior supports previous findings (Agrafiotis et al., 2003), where native users of LSE never looked at the hands, whereas deaf subjects with hearing parents occasionally looked at the hands. However, this difference between native and non-native LSE signers was not relevant when participants perceived SSS, which could suggest that native LSE signers could have automatized the strategy of using peripheral vision when attending LSE, their native natural language, but not when perceiving SSS, despite being quite familiar with this communicative system. Wilbur (2008) argues that manual communication systems, such as SSS, cannot be nativized and acquired as natural languages, as they are artificial systems used to express the spoken language with the hands.

However, the crucial issue is how gaze behavior relates to comprehension. Our results of comprehension did not reveal a superiority of SSS on spoken language in transmitting information, suggesting that despite the tendency to perceive signs peripherally, our participants were not able to fully take advantage from dual streams of information of SSS. One consideration is that the size of the useful visual field can be increased with expertise in a task (Holmqvist et al., 2011), so that when perceiving the LSE, native signers might process more meaningful information from the periphery in a single fixation. These results are coherent with behavioral studies (Parasnis and Samar, 1985; Loke and Song, 1991) that provide evidence for an increased attention in deaf subjects to visual stimuli presented in the periphery. However, the increased spatial attention to periphery might result in a reduced sustained attention to central events (Proksch and Bavelier, 2002), meeting predictions about the balance in visual attention proposed by the division-of-labor hypothesis (Mitchell, 1996). This hypothesis predicts that increased attention to periphery does not necessarily imply an overall increased uptake of information. On the other hand, the study by De Filippo and Lansing (2006) revealed that their participants, who mainly attended the face area when watching video-clips in SSS, were equally likely to make a mistake if the disambiguating information in a sentence came from lip movements or from signs. These results suggest that in that study, participants were able to obtain useful information from both channels of SSS. Future research should explore more deeply the relationship between useful field of view and uptake of information from multiple articulatory channels in deaf perceivers, in order to analyze the respective contribution of speech and signs and the eventual strengthened communication offered by the simultaneity of the two channels in transmitting language.

## Author Contributions

EM: Designed the study, recruited participants and collected data, analyzed and interpreted data, drafted the manuscript. IR-O and DS: Designed the study, interpreted data, revised the manuscript. All authors approved the final manuscript as submitted.

## Conflict of Interest Statement

The authors declare that the research was conducted in the absence of any commercial or financial relationships that could be construed as a potential conflict of interest.

## Footnotes

1 corpus.rae.es/creanet.html
