# Supplementary material for: An Eye Tracking Study on the Perception and Comprehension of Unimodal and Bimodal Linguistic Inputs by Deaf Adolescents
Source: Front Psychol. 2017 Jun 21;8:1044. doi: 10.3389/fpsyg.2017.01044 (PMC5478736; doi:10.3389/fpsyg.2017.01044)

Supplementary Material

An Eye Tracking Study on the Perception and Comprehension of Unimodal and Bimodal Linguistic Inputs by Deaf Adolescents

Eliana Mastrantuono[1]*, David Saldaña[1], and Isabel R. Rodríguez-Ortiz[1]

*** Correspondence:** Eliana Mastrantuono: emastrantuono@us.es

1 Supplementary Table

Table 1s. Example of the stimuli texts and tasks used, in English translation:

Test

A couple of elderly people lived in a street facing the sea. Their house was between the house of a fisherman, on the left, and a park, on the right. The park was in a corner. One night when the spouses were not home, two thieves planned to rob their house. One of the thieves, Peter, got into the house, while the other, Javier, waited outside, hidden in a supermarket in front of the house of the elderly couple. While checking the street, Javier received a call from his girlfriend who was very upset because he had forgotten her birthday. Javier was so distracted apologizing to his girlfriend that he did not see that the elderly couple had come back. They entered the house and found the thief, Peter. The old woman grabbed a broom and ran after Peter down the street, turned the corner of the park and ran towards the north until he reached the police station. There, some agents arrested Peter.

Task 1. Answer the multiple choice questions:

1) What is the most appropriate title for the story?

   a) A family goes to the sea

   b) Bumbling burglars

   c) A village in the mountain

   d) A cheerful elderly couple

2) Two thieves:

   a) Planned to rob the house of a fisherman

   b) Planned to rob the house of old spouses while they were not at home

   c) Both got into the house of the elderly couple when they were sleeping

   d) Planned a robbery in a supermarket

3) The elderly lady:

   a) Chased the burglar she found in her house with a gun

   b) Fainted when she saw a burglar in her house

c) Called the police to arrest the thief

d) Ran after the thief Peter until he reached the police station

Task 2. Find these places in the map:

Elderly couple's house

Fisherman's house

Supermarket

Police Station

Park

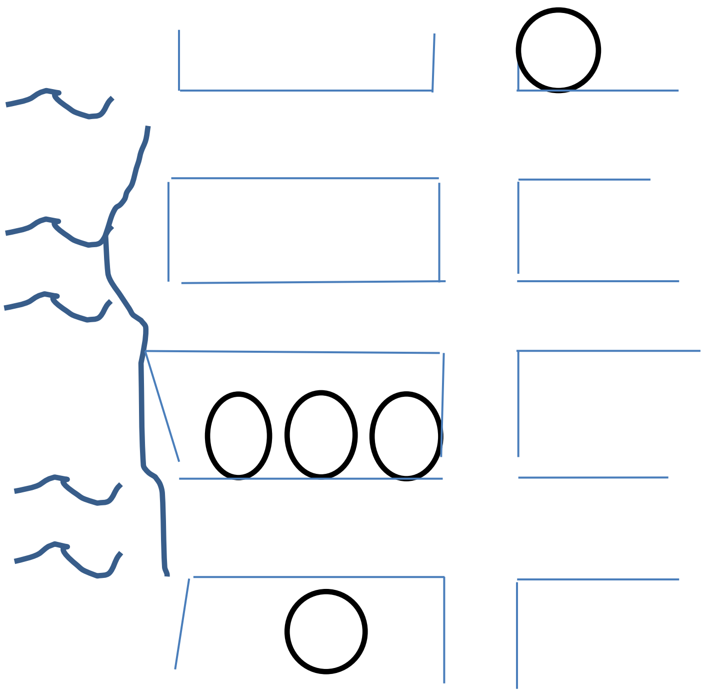

Supplement: Supplementary file 1 [file Data_Sheet_1.pdf]
